# Enhanced Aryltetralin Lignans Production in *Linum* Adventi-Tious Root Cultures

**DOI:** 10.3390/molecules26175189

**Published:** 2021-08-27

**Authors:** Michela Alfieri, Iride Mascheretti, Roméo A. Dougué Kentsop, Roberto Consonni, Franca Locatelli, Monica Mattana, Gianluca Ottolina

**Affiliations:** 1 Institute of Chemical Sciences and Technologies “Giulio Natta”, National Research Council, Via Mario Bianco 9, 20131 Milan, Italy; michela.alfieri@scitec.cnr.it (M.A.); roberto.consonni@scitec.cnr.it (R.C.); 2 Institute of Agricultural Biology and Biotechnology, National Research Council, Via Bassini 15, 20133 Milan, Italy; iride.mascheretti@ibba.cnr.it (I.M.); romeo.dougue@ibba.cnr.it (R.A.D.K.); franca.locatelli@ibba.cnr.it (F.L.); monica.mattana@ibba.cnr.it (M.M.)

**Keywords:** *Linum*, aryltetralin lignans, 6-methoxypodophyllotoxin, elicitor, NMR, methyl jasmonate, coronatine

## Abstract

Lignans are the main secondary metabolites synthetized by *Linum* species as plant defense molecules. They are also valuable for human health, in particular, for their potent antiviral and antineoplastic properties. In this study, the adventitious root cultures of three *Linum* species (*L. flavum*, *L. mucronatum* and *L. dolomiticum*) were developed to produce aryltetralin lignans. The effect of two elicitors, methyl jasmonate and coronatine, on aryltetralin lignans production was also evaluated. The adventitious root cultures from *L. dolomiticum* were obtained and analyzed for the first time and resulted as the best producer for all the aryltetralins highlighted in this system: Podophyllotoxin, 6-methoxypodophyllotoxin and 6-methoxypodophyllotoxin-7-*O*-β-glucoside, the last showing a productivity of 92.6 mg/g DW. The two elicitors differently affected the production of the 6-methoxypodophyllotoxin and 6-methoxypodophyllotoxin-7-*O*-β-glucoside.

## 1. Introduction

Lignans are secondary metabolites widely distributed in higher plants that demonstrate a wide range of biological activities. The main function of these metabolites is to protect plants against herbivores and pathogens and to help their adaptation to adverse environmental conditions. Moreover, most of these compounds also show biological activities including antiviral, cytotoxic, antioxidant, hepatoprotective, anti-inflammatory and antiangiogenic [1,2,3,4]. In particular, the lignans showing cytotoxic activity, such as podophyllotoxin (PTOX) and 6-methoxy-podophyllotoxin (MPTOX), can be used as lead compounds for the development of new therapeutic drug for their anticancer properties [5]. 

The biosynthesis of lignans starts from a regio- and stereoselective, oxidative coupling of two coniferyl alcohol or their biogenetic equivalents (propenyl phenols), to form a large variety of distinct molecules [6,7]. On the base of their chemical structure, they are divided into several classes. The aryltetralin-type lignans (ATLs) have gained attention since the most representative compound, podophyllotoxin, is one of the main precursors of the effective anticancer drugs etoposide, teniposide, GL331, NK611 and TOP-53, which exhibit inhibitory action against the enzyme topoisomerase II [8]. These semisynthetic derivatives are being successfully used to treat several cancers such as lung, testicular, ovarian, stomach and non-Hodgkin lymphoma [9].

Despite the recent advances in the chemical synthesis of the aryltetralin lactone skeleton [10,11,12], the semisynthesis strategy is still preferred to achieve the target drugs. In nature, PTOX is found in the roots and rhizomes of either the *Podophyllum peltatum* and *Podophyllum hexandrum*, but due to overcollection, these species are listed as endangered. In addition to these two recognized producers, there are many other plants being studied for PTOX, or aryltetralin lactone lignans in general, belonging to different clades/families, such as *Laminaceae*, *Apiaceae*, conifer species belonging to *Podocarpus*, *Callitris* or *Juniperus* [13]. Up to now, a very important source of lignans is represented by the genus *Linum*, family *Linaceae*, which includes about 230 species, divided into six sections based on their morphology: *Linum*, *Dasylinum*, *Linastrum*, *Cathartolinum*, *Syllinum* and *Cliococca* [14]. The ATLs are found mainly in the *Syllinum* section [15], where some species are described as PTOX producers with most of the studies related to *L. flavum*, *L. mucronatum* and *L. album*. Lignans are found in the flowering aerial part [16], in seeds [17] and in roots [18].

It has been extensively reported that plant cell cultures can overcome the bottleneck due to the long time required for field cultivation of the plant material, and that metabolite production of these cultures can be induced through elicitor treatments [19]. Elicitors are often used to stimulate the production of plant secondary metabolites in diverse species [15]. In particular, elicitors such as methyl jasmonate (MeJA) or coronatine (COR) have been reported to enhance lignans production in several *Linum* species [20].

However, despite the fact that some biopharmaceutical molecules are produced via cell line cultures [15,21,22], it is generally recognized that some limitations occur, such as low and variable yields of metabolites and genetic instability. The low yield of secondary metabolites in cell cultures has been explained by the lack of cell differentiation. Then, an alternative strategy to cell culture is the organized tissue culture of roots or shoots [23]. A wide range of natural products has been produced using adventitious and hairy-root cultures, including lignans, exploiting their high genetic stability, biosynthetic capabilities, biomass production and the possibility to be used for several successive generations [24]. These tissues can be cultivated in large-scale bioreactors for commercial production of bioactive compounds and can be elicited to obtain a higher yield. Until recently, genetically transformed hairy roots had received more attention for phytochemicals production than adventitious roots. However, the adventitious roots cultures (ARc) are emerging since they are considered the more natural material (untransformed) thus leading to their high commercial value compared to hairy roots.

In this study, *L. flavum*, *L. mucronatum* and *L. dolomiticum* ARc were obtained and the effect of two elicitors, MeJA and COR, was investigated on ATLs production and compared among these species for the first time. The antioxidant capacity, total phenols and flavonoids content of root extracts were also assessed. The ATLs were purified by preparative TLC and HPLC and structurally determined by NMR.

## 2. Results

### 2.1. ARc Production and Effect of Elicitation Treatments 

**Figure 1:**
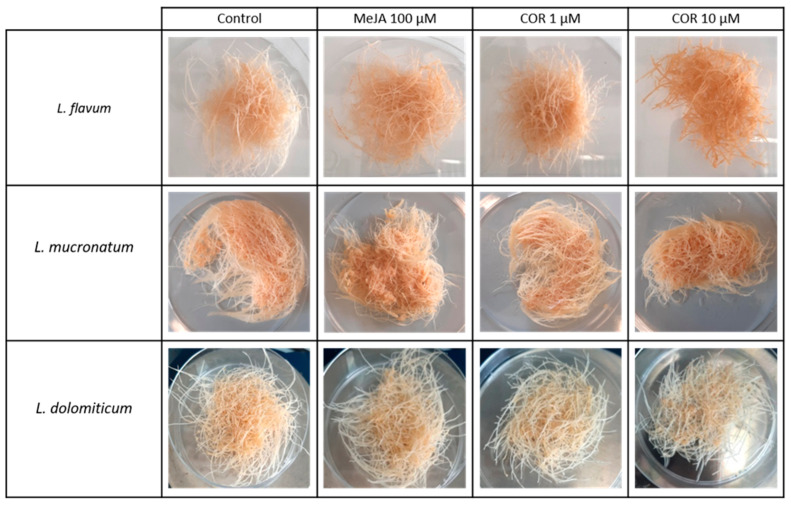
ARc from *L. flavum*, *L. dolomiticum* and *L. mucronatum* grown for 25 days on MS-II medium with or without elicitors: 100 µM MeJA, 1 µM COR, 10 µM COR.

ARc were obtained from three *Linum* species: *flavum*, *mucronatum* and *dolomiticum*. All the ARc obtained from the three species showed both good stability and biomass production, referred to as FW, over a 25-day growth period (Figure 1).

**Figure 2:**
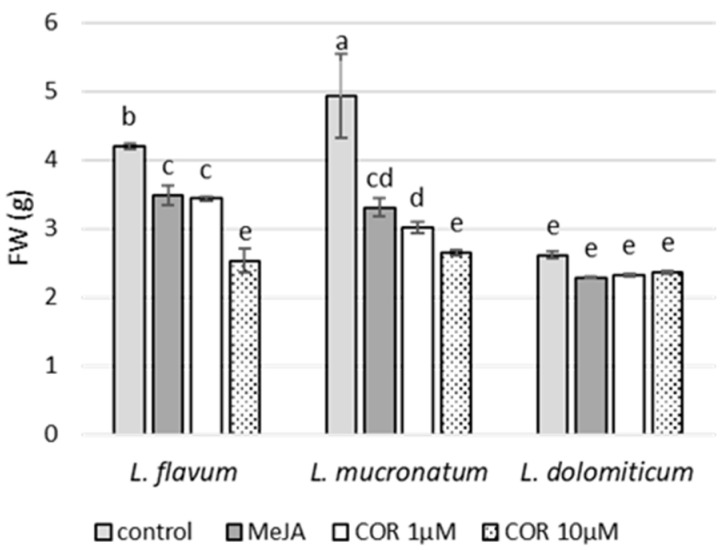
Effect of elicitors on the growth of ARc of *L. flavum*, *L. mucronatum* and *L. dolomiticum* over a cultivation period of 25 days in MS-II liquid medium expressed as fresh weight (FW) in grams; control (light gray), MeJA (100 µM, dark gray), COR 1 µM (white), COR 10 µM (dotted). Each value is the mean of three biological replicates ± SD. The samples with different letters are significantly different at *p* ≤ 0.05 according to Duncan test. The growth index of each ARc culture is reported from left to right; *L. flavum*: 4.2, 3.4, 3.3, 2.2; *L. mucronatum*: 5.2, 3.1, 2.8, 2.3; *L. dolomiticum*: 2.3, 1.9, 1.9, 2.0.

This growing time was adopted since the three *Linum* ARc needed almost 8–10 days of adaptation to the liquid medium before entering exponential growth. Then, on day 21 MeJA and COR treatments were applied to the cultures for four days. The use of MeJA and COR on the ARc of the species considered in this work is reported for the first time, even though their use was already reported for cell suspensions and ARc of other *Linum* species. As shown in Figure 1 and Figure 2, the elicitor treatments caused an inhibition of growth as already reported for other species [25]. A two-way analysis of variance (ANOVA, Table S1) highlighted a reduction on the final growth of *L. flavum* and *L. mucronatum* ARc after the addition of MeJA or COR (Figure 2). On the contrary, in *L. dolomiticum* ARc, MeJA and COR did not significantly inhibit growth (Figure 2).

### 2.2. Total Phenols, Total Flavonoids Content and DPPH Radical Scavenging Activity

Before estimating the ATLs content, the total phenols, the total flavonoids and the antioxidant capacity of the ARc extracts were investigated in function of the two elicitors MeJA and COR. 

**Figure 3:**
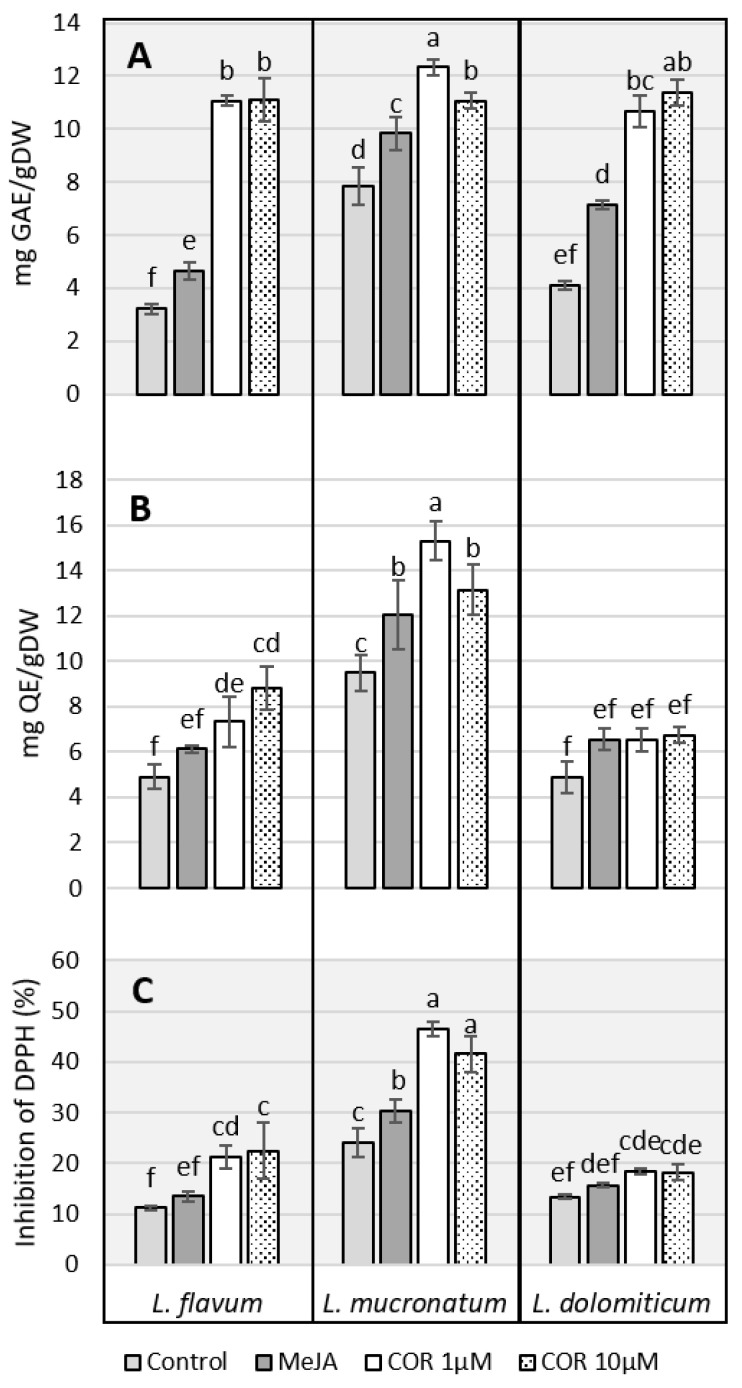
Effect of elicitors on *L. flavum*, *L. mucronatum* and *L. dolomiticum* ARc; control (light gray), MeJA (100 µM, dark gray), COR 1 µM (white), COR 10 µM (dotted). (**A**) Phenol content expressed as gallic acid equivalent, mg GAE/g DW. (**B**) Flavonoid content expressed as quercetin equivalent, mg QE/g DW. (**C**) Antioxidant capacity expressed as percent of inhibition of DPPH. Each value is the mean of three biological replicates ± SD. The samples with different letters are significantly different at *p* ≤ 0.05 according to Duncan test.

The results were analyzed using two-way analysis of variance (ANOVA, Table S1), a pairwise comparison test (Tukey’s HSD, Figures S6–S8) and a multiple-comparison test (Duncan test, Figure 3) in order to compare three different species of *Linum* and four different treatments on the accumulation of phenols, flavonoids and antioxidant capacity. Both factors (species and elicitor treatments) and their interaction were found to be significant (Table S1). The total phenol content, expressed as gallic acid equivalent (GAE) (Figure 3A), indicated a marked influence of the elicitor in all the three ARc species. In *L. flavum* treated with COR at both concentrations, the production of phenols was nearly three times compared to the control or to MeJA. In *L. dolomiticum*, MeJA nearly doubled the amount of total phenol compared to the control, and COR had an even stronger effect. The control samples of *L. mucronatum* showed a higher level of phenols than the other species and the increase due to the elicitation was less prominent. It is noteworthy that the maximum amount of total phenol reached after COR elicitation was similar regardless to the plant species. The flavonoid content was measured as quercetin equivalent (QE) (Figure 3B). As for total phenols, the flavonoid level was enhanced by COR more than MeJA apart from *L. dolomiticum* where the increase due to the elicitors was flattened (Figure 3B). 

The antioxidant capacity evaluated as the scavenging of DPPH free radicals is reported in Figure 3C. Elicitors were found to increase the antioxidant capacity of the ARc, with COR showing a stronger effect than MeJA, especially in *L. flavum* and *L. mucronatum*. *L. dolomiticum* showed minor differences among the treatments. In general, the elicitation due to COR was stronger than MeJA, as well as at lower concentration (1 µM). It seems that the best match between elicitor concentration and biomass growth inhibition in our ARc is reached with 1 µM COR. The reaction of DPPH with the ARc extracts was also performed on a TLC plate after chromatographic separation (Figure S1). The result obtained confirms that *L. mucronatum* ARc has the strongest DPPH radical scavenging activity as highlighted in Figure 3C and suggests that this activity is mainly due to the presence of a specific compound with R_f_ 0.53 that was not detectable in ARc extracts from *L. flavum* and *L. dolomiticum*.

### 2.3. NMR Identification of ATLs

**Figure 4:**
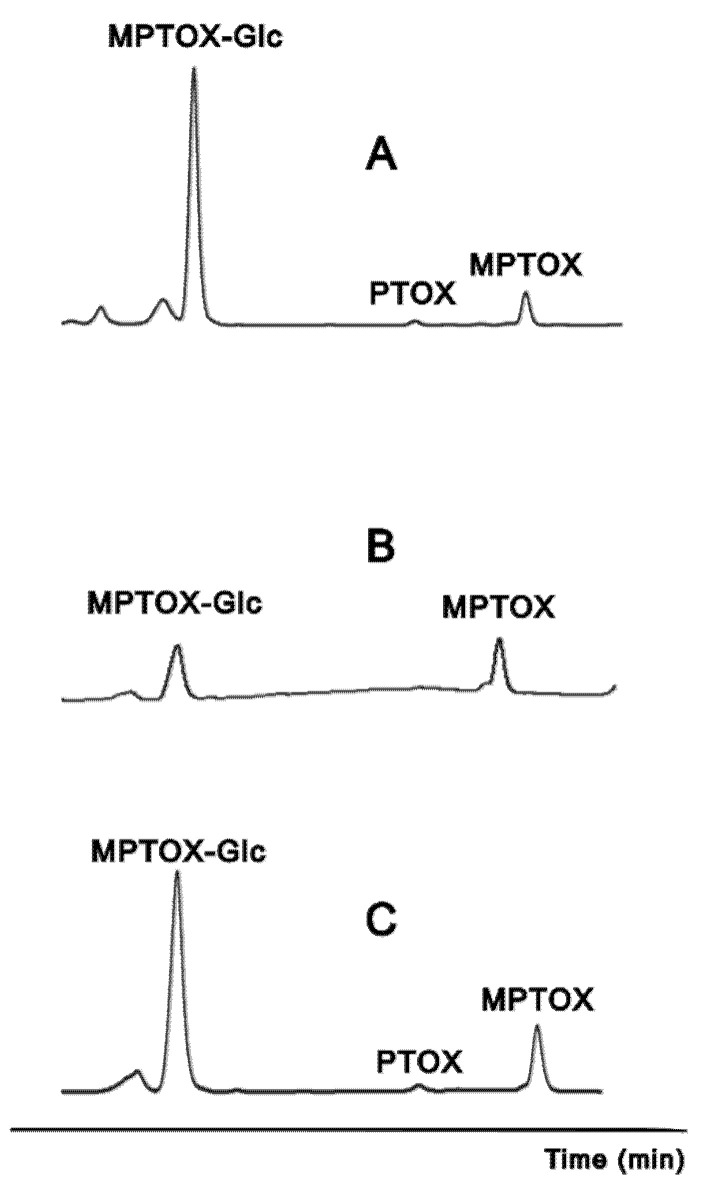
HPLC chromatograms of the ATLs portion, 18 to 24 min at 254 nm, COR-treated extract from (**A**): *L. flavum*, (**B**): *L. mucronatum* (**C**): *L. dolomiticum*. MPTOX–Glc (R_t_ 18.8 min), PTOX (R_t_ 22.2 min) and MPTOX (R_t_ 23.9 min) were identified. See also Figure S2 for the whole chromatogram.

The accumulation of ATLs in the ARc from *L. flavum*, *L. mucronatum* and *L. dolomiticum* was studied. The typical HPLC chromatogram profile of the adventitious root extracts is reported in Figure 4 for the aryltetralin lignan portion centered on PTOX retention time (see Figure S2 for the whole chromatogram). The amount of PTOX, R_t_ 22.2 min, was very small compared to other compounds, and below the limit of detection (LOD) in *L. mucronatum*. In the same portion of the HPLC profile, two other signals were recorded at R_t_ of 18.8 and 23.9 min. These molecules were purified by means of TLC and HPLC and identified by NMR (Figures S3 and S4). 

Two molecules were purified by preparative TLC of the *L. flavum* sample. The ^1^H-NMR spectra revealed the signals of 6-methoxypodophillotoxin (Figure S3) and the signals corresponding to 6-methoxypodophyllotoxin-7-*O*-β-glucoside (Figure S4), in full agreement with previous NMR assignments of these molecules [26].

### 2.4. Quantitative Analyses of ATLs

**Figure 5:**
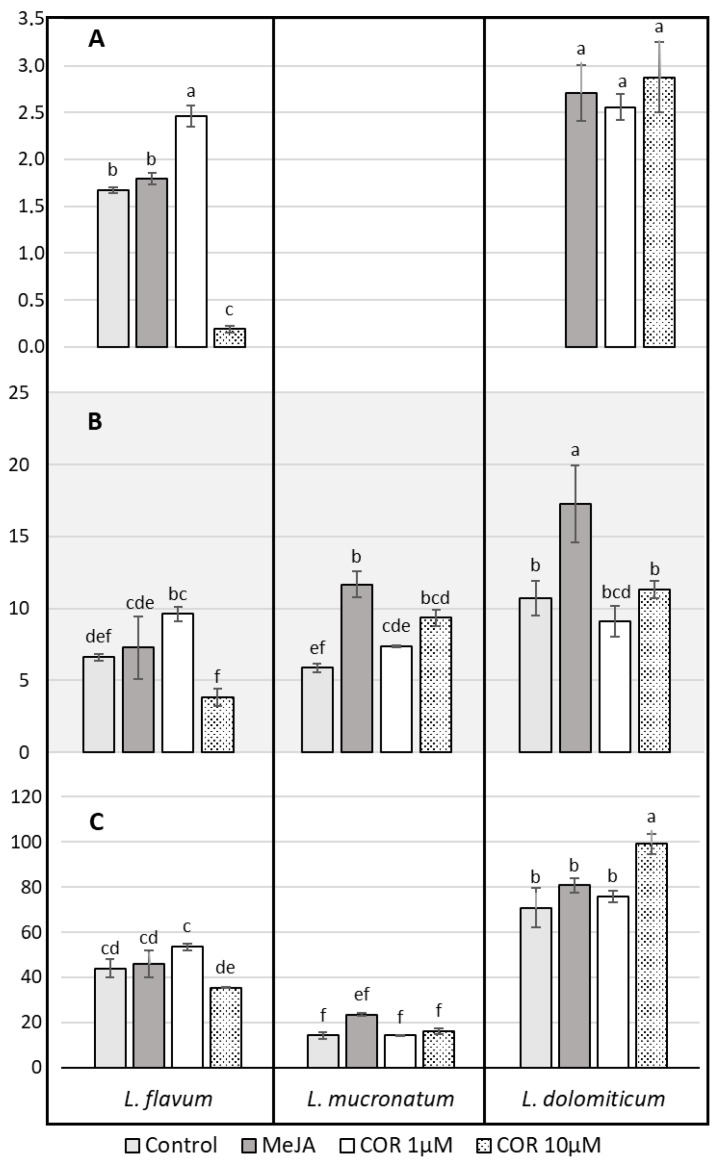
ATL content (mg/g DW): (**A**): PTOX; (**B**): MPTOX; (**C**): MPTOX–Glc. Control (light gray), MeJA (100 µM, dark gray), COR 1 µM (white), COR 10 µM (dotted). Each value is the mean of three biological replicates ± SD. The samples with different letters are significantly different at *p* ≤ 0.05 according to Duncan test.

The compounds identified by ^1^H-NMR were quantified in all the ARc samples, the control and those treated with the elicitors. The results obtained after 25 days of culture are reported in Figure 5. A statistical analysis was applied using two-way ANOVA (Table S2), a pairwise comparison test (Tukey’s HSD, Figures S9–S11) and a multiple-comparison test (Duncan test, Figure 5) to compare the effect of the species and the elicitor treatments. Both factors, species and treatments, and their interaction were found to be significant for the accumulation of PTOX and MPTOX (Table S2). Conversely, the elicitor treatment was not significant for the accumulation of MPTOX–Glc. Among the three *Linum* species, PTOX was the least abundant lignan reaching 1.67 mg/g DW in *L. flavum* ARc with an increase after 1µM COR elicitation up to 2.46 mg/g DW and becoming negligible in the presence of 10 µM COR (Figure 5). In the case of *L. mucronatum* ARc, PTOX was not detected even under elicitation. The production of PTOX by *L. dolomiticum* ARc was 2.55 mg/g DW under 1 µM COR elicitation, being almost undetectable in control samples (Figure 5). Conversely, the most represented compounds present in the ARc of the three *Linum* species were MPTOX and MPTOX–Glc (Figure 5). 

The highest productive species in terms of MPTOX was *L. dolomiticum* with 10.7 mg/g DW with an increase up to 17.2 mg/g DW after MeJA elicitation. The production of MPTOX–Glc, (Figure 5) showed large differences among the ARc of the three species. In detail, *L. flavum* reached 44.0 mg/g DW with a slight but not significant increase after elicitation, while *L. mucronatum* showed the lowest production with 14.3 mg/g DW in the control condition with an increase to 23.4 mg/g DW after MeJA elicitation. The most productive species was *L. dolomiticum* with an MPTOX–Glc production of 70.8 mg/g DW rising to 92.6 mg/g DW after 10 µM COR elicitation. Our results indicate ARc of *L. dolomiticum* as the best producer among the three species investigated. 

The culture media were also analyzed by HPLC to verify whether the lignans produced were excreted in the medium; however, the presence of PTOX, MPTOX or MPTOX–Glc were not detected.

To clarify the relationships among all the compounds analyzed in response to the elicitor treatments, the Pearson correlation was performed. This analysis (Figure S12) highlighted, for each treatment, that there is a high significant negative correlation between the groups of the aryltetralins and the phenols, flavonoids and antioxidant activity. These correlations were particularly strong for the COR 1 µM, followed by MeJA and COR 10 µM.

## 3. Discussion

The presence of ATLs (podophyllotoxin-like molecules) have been described in the *Syllinum* section of the genus *Linum*, mainly in the species *L. flavum*, *L. mucronatum*, *L. album* and *L. nodiflorum* [15]. Cell suspension or callus cultures as well as hairy roots of such species have been produced and shown to accumulate different ATLs, with the hairy roots being the most productive tissue [27,28,29]. Among the various tissue cultures, ARc represent one of the best choices for secondary metabolite production especially because they do not need genetic modification, making them safer [30]. In this context, ARc was obtained from three *Linum* species: *flavum*, *mucronatum* and *dolomiticum*. ARc was established for *L. dolomiticum* and *L. mucronatum* for the first time, whereas root cultures of *L. flavum* were already realized [29]. In particular, *L. dolomiticum* was chosen for its endemic origin (linked to dolomite rock grassland) since it is well known that variability in secondary metabolite production exists among species grown in particular habitats [31]. The ARc obtained from the three species revealed good stability and good biomass production over a period of 25 days of growth. Elicitation is a well-described method to enhance secondary metabolites production by tissue cultures. In particular, MeJA and COR represent two hormonal elicitors with a demonstrated role in increasing secondary metabolites synthesis through signal transduction by activating key differentially expressed genes (DEGs) in biosynthetic pathways from the secondary metabolite of interest [25]. The use of MeJA and COR on the ARc of the species considered in this work is reported for the first time, whereas several studies describe their use on cell suspensions, adventitious or hairy roots of other *Linum* species [32,33,34]. As reported for other species, the elicitor treatment affected ARc growth differently in the three species considered: A marked inhibitory effect was observed for *L. flavum* and *L. mucronatum* ARc and no effect was evident for *L dolomiticum ARc.* Regarding the effect of elicitors on phenols and flavonoids accumulation and on the antioxidant capacity, COR seems to be more effective than MeJA. However, in the case of *L. dolomiticum* Arc, the flavonoids and antioxidant capacity was almost unaffected by the treatments. The fact that there is not a striking difference between the two different concentrations of COR suggest the presence of a threshold concentration common to all the species analyzed. Furthermore, looking at the absolute concentrations, there seems to be a trade-off between growth and productivity. Indeed, it has been reported that lower elicitor concentrations result in better biomass growth and productivity of the culture (i.e., paclitaxel production) [35]. The production of ATLs lignans has been described for several *Linum* species from the section *Syllinum* both from cell suspension and hairy roots [36]. In particular, for hairy roots, a production of 0.8 mg/g DW PTOX has been described, while for *L. flavum* hairy roots, that was about twice that of the corresponding cell suspension with a production of 0.8 mg/g DW PTOX [36]. As far as the *L. flavum* whole plant is concerned, variations in ATLs content are described among the different organs, PTOX and 6-MPTOX are present in all plant organs, but a higher concentration of PTOX (0.27–0.33%) than 6-MPTOX (0.04–0.12%) has been found in extracts of upper plant parts, petals, leaves and productive organs [17]. However, root tissue cultures usually show higher concentrations than whole plant organs. Actually, our adventitious roots production was greater than *L. flavum* hairy roots, being twice as high in control conditions and three times higher after 1 mM COR elicitation. These results suggest that adventitious roots could represent a very promising tissue for PTOX production. In contrast, in L. mucronatum ARc, PTOX was not detected even under elicitation.

This result is opposed to that reported by Samadi for *L. mucronatum ssp*. *mucronatum* hairy roots [37] reporting a production of 5.78 mg/g DW PTOX. This discrepancy could be due to the origin of *L. mucronatum* seeds used [37], which were collected from the mountain region of Iran, at an altitude of 1800 m, with respect to our seeds that were from the Turkey region. Different climatic and pedologic environment could determine differences in biochemical traits [31]. 

The proposed biosynthetic pathways leading to podophyllotoxin (PTOX) and 6-methoxypodophyllotxin (MPTOX) and their glucosides starts from a common precursor, (-)–deoxypodophyllotoxin, which can be directed to the synthesis of PTOX by DOP7H (deoxypodophyllotoxin 7-hydroxylase) [32] or to MPTOX by DOP6H (deoxypodophyllotoxin 6-hydroxylase) then to the corresponding glycosylate MPTOX–Glc [38]. Our data suggest that the second biosynthetic route is preferred in the ARc obtained from the three species, and that in all the experiments, the amount of β-peltatin, the precursor of MPTOX, was negligible. Despite this fact, our data demonstrate that *L. dolomiticum* was the highest productive species in terms of MPTOX with 10.7 mg/g DW with an increase up to 17.2 mg/g DW after MeJA elicitation, which is approximately the same amount of MPTOX produced in *L. album* ARc elicited with 1µM COR [32]. A recent study has demonstrated the cytotoxic activity of MPTOX toward four selected human carcinomas and against a normal fibroblast cell line and was described its mechanism of action [39], which is comparable to PTOX. Due to its antiproliferative activity, this molecule could be of interest to produce new anticancer drugs and to bypass the resistance mechanisms against podophyllotoxin-derived drugs [40]. The most striking result was the production of MPTOX–Glc by *L. dolomiticum* ARc, 70.8 mg/g DW rising to 92.6 mg/g DW after 10 µM COR elicitation. This value is one of the highest reported for this molecule even when compared to hairy roots of other species [36,37,41]. In any case, the hairy roots are not always the most productive tissue as regards these molecules, as reported for PTOX and MPTOX in adventitious and hairy roots of *L. album* [32]. Our results indicate the ARc of *L. dolomiticum* as the best producer among the three species investigated. 

The culture media were also analyzed by HPLC to verify whether the lignans produced were excreted in the medium, as reported for some *Linum* species in a very low amount [25,36]; however, the presence of PTOX, MPTOX or MPTOX–Glc were not detected. The digestion of MPTOX–Glc with β-glucosidase showed that this molecule can be easily converted to MPTOX. In a scale-up perspective, the production of MPTOX could be obtained through *L. dolomiticum* ARc, which produces good quantities of MPTOX and MPTO–Glc, and then the extract can be treated with β-glucosidase to obtain MPTOX if needed. The negative correlation among phenols, flavonoids and ATLs in response to the elicitor treatments suggest that the elicitation activates different pathways including those of lignans or others producing secondary metabolites belonging to the classes of phenols and flavonoids. For this reason, the ARc of *L. mucronatum*, accumulating more phenols and flavonoids compounds, is less suitable to produce ATLs, whereas *L. dolomiticum* and *L. flavum* ARc seem to be more performing. Regarding the use of elicitors to increase the ATLs production in *Linum*, we can conclude that both MeJA and COR were appropriate to activate the lignans pathway, as previously reported for other *Linum* species [32,33,34]. The same elicitors were also able to enhance the arylnaphtalenic production in *L. austriacum* [42]. Our studies showed that MeJA is more effective on *L. mucronatum* ARc and 1µM COR on *L. flavum* ARc. In *L. dolomiticum* ARc, MeJA enhanced the MPTOX production whereas COR showed a better effect on MPTOX–Glc. These data confirm that signal molecules are effective in enhancing secondary metabolisms, but the effect of each elicitor, even if belonging to the same class of compound, could be different on the same species. The results obtained indicate that ARc could be a very promising tissue for secondary metabolites production especially when using the proper elicitor. This technology could have commercial applications through a scale-up process in bioreactors as reported for some species such as *Hypericum perforatum* and *Polygonum multiflorum* [43]. The main advantage for the use of ARc with respect to hairy roots is the absence of opine-like substrates (dangerous to mammalian cells) that are usually produced as byproducts of the hairy root system [44]. In general, the results reported point out that ARc represents a very good system for industrial production of bioactive compounds for their better biosynthetic ability and a great stability. Moreover, ARc could be preferred to hairy roots since they are simpler to obtain and safer.

## 4. Materials and Methods

### 4.1. Chemicals

Methyl jasmonate (MeJA), coronatine (COR), the Folin–Ciocalteu reagent, 2,2-Diphenyl-1-picrylhydrazyl (DPPH), triphenyl tetrazolium chloride (TTC), Murashige and Skoog Basal Medium (MS), sucrose, gallic acid, quercetin, podophyllotoxin, β-glucosidase, indole-3-acetic acid (IAA), indole-3-butyric acid (IBA), α-naphthalene acetic acid (NAA) and salts were purchased from Merck (Darmstadt, Germany); the solvents were from Honeywell (Milan, Italy); the pre-coated TLC-plates SIL G-100/UV254 were from Macherey-Nagel (Dueren, Germany); water (purified water) was obtained from MilliQ (Millipore, Darmstadt, Germany).

### 4.2. Plant Material 

Seeds of *L. flavum* and *L. dolomiticum* were purchased by Jelitto (Jelitto Staudensamen, Germany) and *L. mucronatum* was obtained from USDA (U.S. Department of Agriculture, Beltsville, MD, USA). The seeds were surface sterilized in 70% ethanol for 1 min, then in a solution of sodium hypochlorite 1% (*v*/*v*) for 5 min, washed 5 times with sterilized water and then germinated in MS basal medium [45] at 22 °C in dark conditions. After one week, the seedlings were placed at 25 °C, for one month, under 16 h of light and 8 h of darkness.

### 4.3. Establishment of ARc and Elicitor Treatments

#### 4.3.1. ARc Induction

ARc was generated from leaves collected from one-month-old plantlets of *L. flavum*, *L. dolomiticum* and *L. mucronatum*. The sterile leaf explants were laid horizontally on Petri dishes with solid (0.8% agar) MS supplemented with vitamins, 2 mg/L α-naphthalene acetic acid (NAA), 0.4 mg/L kinetin and 30 mg/L of sucrose. The pH was adjusted to 5.8 using a KOH solution. After 4 weeks, the length of the root segments was 0.8–1.3 cm. In order to increase the biomass production, roots were individually transferred to half-strength MS medium supplemented with 20 mg/L of sucrose 0.5 mg/L indole-3-butyric acid (IBA) and 0.1 mg/L indole-3-acetic acid (IAA) (shortened from now on as MS-II) and the roots were sub-cultured every month. All the ARc lines were maintained on solid agar medium as a starter material to be used for new liquid cultures.

#### 4.3.2. Elicitor Treatments

For the elicitation treatment, 0.8 g of ARc of the three *Linum* species were transferred into a 250 mL Erlenmeyer flask containing 20 mL of MS-II liquid medium and were grown for 21 days at 23 °C on an orbital shaker (110 rpm) in dark condition and the medium was renewed each week. The stock solution of MeJA, dissolved in ethanol, and COR dissolved in water were prepared and added to the final concentration of MeJA 100 µM, COR 1 µM and 10 µM, then left four days. Ethanol was added to the control samples. 

#### 4.3.3. ARc Growth and Viability

Root growth was measured as fresh weight (FW). Roots were harvested and washed three times with sterilized water, dried on filter paper to remove all external moisture and weighed. For the determination of root viability, a solution of 10 mM KH_2_PO_4_, 3% (*w*/*v*) sucrose and 0.25% (*w*/*v*) triphenyl tetrazolium chloride (TTC) was used as a visual indicator of root viability [46]. The growth of roots was monitored up to 25 days. 

### 4.4. Extraction 

Extraction was performed from powdered lyophilized tissues. The samples were dispersed in a hydroalcoholic solution (methanol 80% *v*/*v*) placed in a rotary shaker at 20 °C overnight, and finally centrifuged for 5 min at 13,000× *g*. The extraction was repeated twice. The clear supernatants were collected and used for total phenolics and flavonoids determination, DPPH radical scavenging assay and chromatographic analysis. The culture medium was extracted with 4 × 5 mL CHCl_3_, and then the organic layers were combined, filtered and dried.

### 4.5. Total Phenols, Total Flavonoids Content and DPPH Radical Scavenging Activity 

#### 4.5.1. Total Phenols Content

The amount of total phenol was evaluated by the Folin–Ciocalteu assay reagent according to Ainsworth et al. [47] using gallic acid as a standard. Briefly, 50 µL of the 80% (*v*/*v*) methanol extract solution was mixed with 750 µL of water and 50 µL of 10-fold diluted Folin–Ciocalteu reagent and allowed to stand for 10 min at room temperature; then 150 µL of the 20% (*w*/*v*) sodium carbonate solution was added to each sample and placed in the dark at room temperature for two hours. The absorbance was spectrophotometrically measured at 765 nm. Total phenols were expressed as milligrams of gallic acid equivalent per gram of DW (mg GAE/g DW). The calibration curve range was 0–6 µg/mL.

#### 4.5.2. Total Flavonoids Content

The total flavonoids content was measured following the method of Wolfe et al. [48]. A total of 50 μL of the sample diluted with 1.45 mL of water was mixed with 75 μL of 5% sodium nitrite and incubated for 5 min followed by 150 μL of 10% aluminum chloride for 6 min. Finally, 500 μL of 1M NaOH was added, and the mixture was adjusted to 2.5 mL with water. The absorbance versus the prepared blank was spectrophotometrically measured at 425 nm after 10 min. The total flavonoids contents of the different treatments were expressed as milligrams of quercetin equivalents per gram of dry matter (mg QE/g DW). The calibration curve range was 0–3.2 µg/mL. 

#### 4.5.3. DPPH Radical Scavenging Activity 

The free radical scavenging activities of plant extracts were determined using DPPH free radical assay [49] using 50 µL of each sample extract and 2950 µL of 80% aqueous-methanolic 0.102 mM DPPH. After 15 min the absorbance reduction was spectrophotometrically measured at 515 nm. The ability to scavenge DPPH was calculated as follows:

(%) DPPH radical scavenging activity = [(Act − Asa)/Act] × 100(1)

where Act is the absorbance of DPPH radical plus methanol and Asa is that of DPPH radical plus the sample extract. Radical scavenging activity is shown as the percentage of DPPH inhibition for mg of DW.

#### 4.5.4. HPTLC-DPPH Test

TLC-DPPH test was used to screen the presence of free radical scavenger spots. Each extract (control, MeJA and 1 µM COR) was applied on HPTLC Silica Gel 60 F254 aluminum sheets with a distance of 4 mm between them using Linomate 5 controlled by winCATS software version 3.0. The plate was developed, to a distance of 90 mm, in a vertical chamber pre-saturated for 20 min with a mobile phase for phenolic acid composed of chloroform/ethyl acetate/acetone/formic acid (4:3:2:1), then dried and observed at 366 nm [50]. The plate was sprayed with DPPH 0.05% in methanol and kept in dark conditions for 30 min. The radical scavenging activity was detected by the discoloration of DPPH.

### 4.6. HPLC Analysis

HPLC analysis was carried out using Jasco instruments equipped with a photodiode array detector. Separation was performed using a Synergi polar RP 80 Å (250 mm × 4.60 mm, 4 μm, Phenomenex) and a gradient system consisting of water (A) and acetonitrile (B) as follows: 0–20% B for the first 15min, 20–70% B from 15 to 25 min, 70% B for 3min and 70–0% B up to 30 min. The flow rate was 1.0 mL/min, the injection volume was 50 µL and the wavelength used for the integration of the signals was 250 nm. Quantification was performed using calibration curves of each standard as reported in Table 1.

### 4.7. Preparative TLC, Semi-Preparative HPLC

The roots collected after 25 days were used for lignans extraction as previously described. The organic fraction was subjected to preliminary preparative TLC separation (mobile phase CH_3_OH–CHCl_3_). The major bands were collected (MPTOX Rf 0.24 (1/99 *v*/*v*); MPTOX–Glc Rf 0.59 (15/85 *v*/*v*) and subjected to purification by means of semi-preparative HPLC. Separation was carried out with Synergi hydro RP 80 Å (250 mm × 10 mm, 4 μm, Phenomenex) using water and acetonitrile as eluents. The MPTOX–Glc and MPTOX obtained reached a purity up to 95%.

### 4.8. NMR Identification of MPTOX and MPTOX–Glc

Purified compounds have been dissolved in deuterated chloroform (99.98% of 2H) and spectra recorded on a Bruker DMX 600 spectrometer, equipped with a 5 mm reverse probe with a z gradient coil. All spectra were recorded at 25 °C and referenced to the solvent signal for both proton and carbon chemical shifts, occurring at 7.27 ppm and 77.2 ppm, respectively. Monodimensional ^1^H, bidimensional ^1^H–^13^C HSQC and HMBC spectra were recorded with 8000 Hz of sweep width over 64k and 2k data points for monodimensional and bidimensional experiments, respectively.

### 4.9. Digestion of MPTOX–Glc with β-Glucosidase

An aliquot of 85 µg of purified MPTOX–Glc was digested with 20 U of β-glucosidase from almonds. The reaction was carried out in a phosphate buffer pH 5.0 at 25 °C for 24 h. The digested extracts were analyzed by HPLC at 2, 4, 6 and 24 h. 

### 4.10. Statistical Analysis

The results were subjected to statistical analysis by means of R (see Statistical Report in the Supplementary Materials).

## Figures and Tables

**Table 1:** HPLC calibration curve data.

Analytes	R_t_ (min)	Concentration Range (mg/L)	Regression Equation	Correlation Coefficient (R^2^)	LOD (mg/L)	LOQ (mg/L)
PTOX	22.2	250–1700	y = 639956.6x + 625206.9	0.9999	20.2	67.3
MPTOX	23.9	107–533	y = 509126.1x − 508145.6	0.9985	23.1	76.9
MPTOX―Glc	18.8	125–1000	y = 334707.7x − 586706.3	0.9994	32.7	109.0

## Data Availability

Not applicable.

## Supplementary Materials

The following are available online, Figure S1: TLC plate of lignans extracts after reaction with DPPH, Figure S2: HPLC chromatogram, Figures S3 and S4: NMR spectra. Statistical Report. Table S1: Two-way ANOVA of growth, phenols, flavonoids and antioxidant capacity, Figures S5–S8: Tukey HSD post hoc test for growth, Table S2: Two-way ANOVA on ATLs, Figure S9: Tukey HSD post hoc test for PTOX, Figure S10: Tukey HSD post hoc test for MPTOX, Figure S11: Tukey HSD post hoc test for MPTOX―Glc, Figure S12: Correlation graphs.

## Sample Availability

Not available.
