# Supplementary material for: Enhanced Aryltetralin Lignans Production in Linum Adventi-Tious Root Cultures
Source: Molecules. 2021 Aug 27;26(17):5189. doi: 10.3390/molecules26175189 (PMC8434161; doi:10.3390/molecules26175189)

Enhanced aryltetralin lignans production in *Linum* adventitious root cultures

Michela Alfieri[1], Iride Mascheretti[2], Roméo A. Dougué Kentsop[2], Roberto Consonni[1], Franca Locatelli[2], Monica Mattana[2] and Gianluca Ottolina[1,*]

Supplementary Materials:

Table of contents

1. TLC plate of lignans extracts after reaction with DPPH (**Figure S1**)

2. HPLC chromatogram (**Figure S2**)

3. NMR spectra (**Figures S3**, **S4**)

4. Statistical Report
   4.1 Two-way ANOVA on growth, phenols, flavonoids, and antioxidant capacity (**Table S1**)
   4.2 Pairwise comparison growth, phenols, flavonoids, and antioxidant capacity (**Figures S5, S6, S7** and **S8**)
   4.3 Two-way ANOVA on ATLs (**Table S2**)
   4.4 Pairwise comparison on ATLs (**Figures S9**, **S10** and **S11**)
   4.5 Pearson correlation (**Figure S12**)

1. TLC plate of lignans extracts after reaction with DPPH (Figure S1)

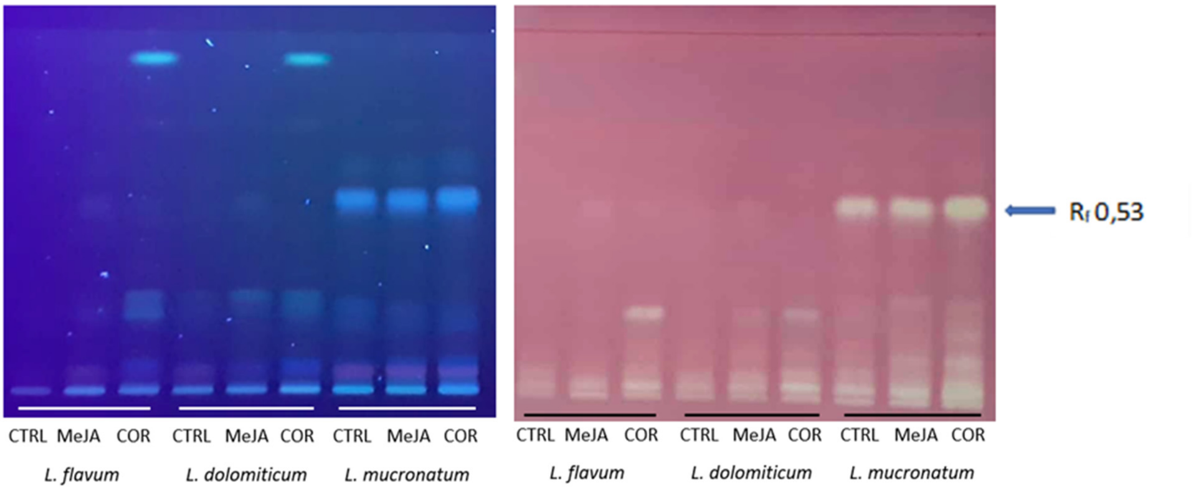

Figure S1. TLC plate of lignans extracts of the *Linum* species at 366 nm (left), and after reaction with DPPH (right).

2. HPLC chromatogram (Figure S2)

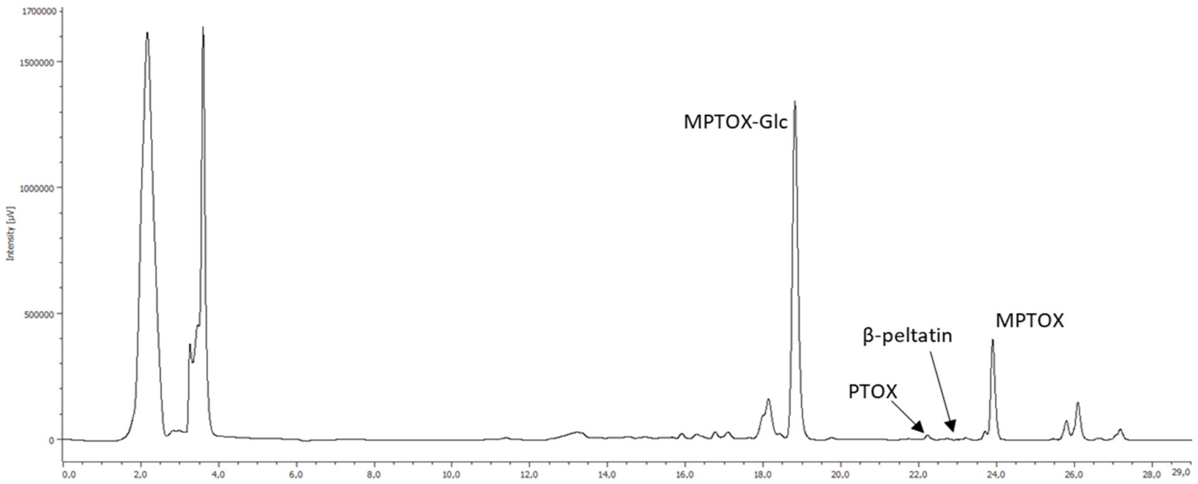

Figure S2 HPLC chromatograms from *L. dolomiticum* COR-treated extract. MPTOX—Glc (Rt 18.8 min), PTOX (Rt 22.2 min) and MPTOX (Rt 23.9 min) were identified. The peak corresponding to β-peltatin was no found.

3. NMR spectra (Figures S3, S4)

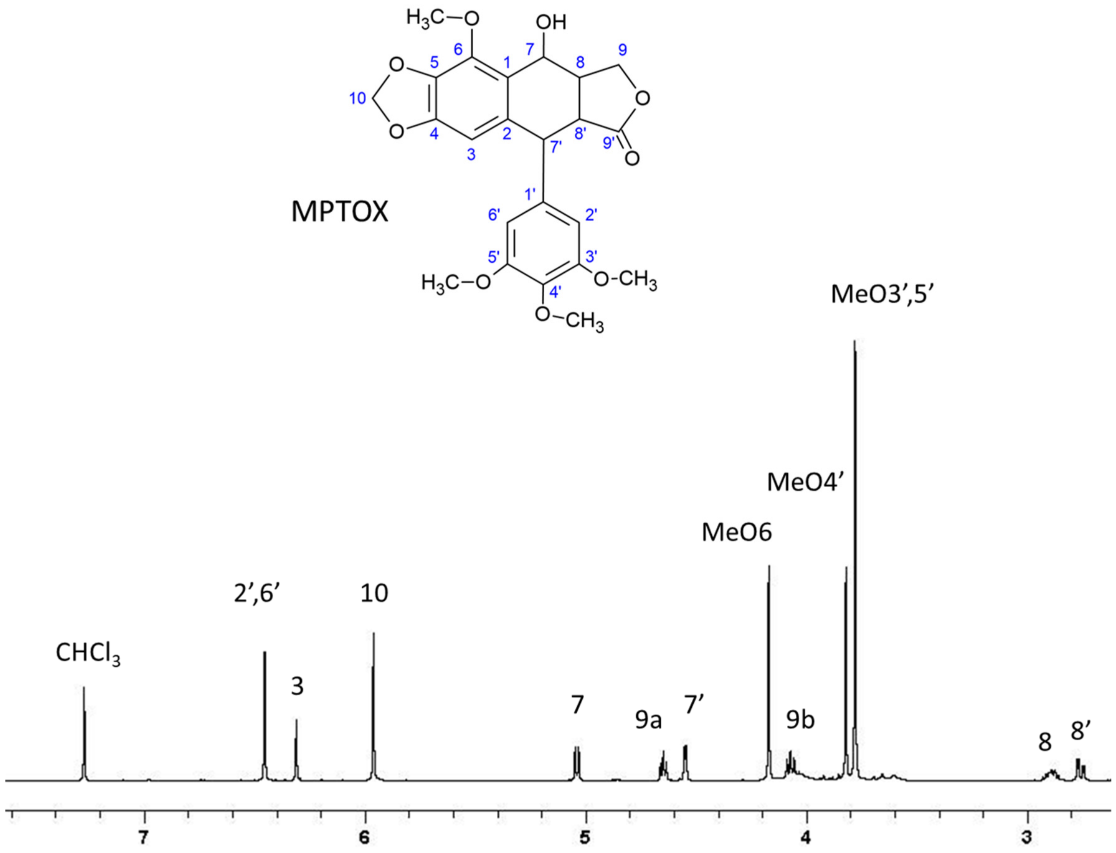

Figure S3 ^{1}H NMR spectra obtained from *L. flavum* sample with signals corresponding to 6-methoxypodophyllotoxin

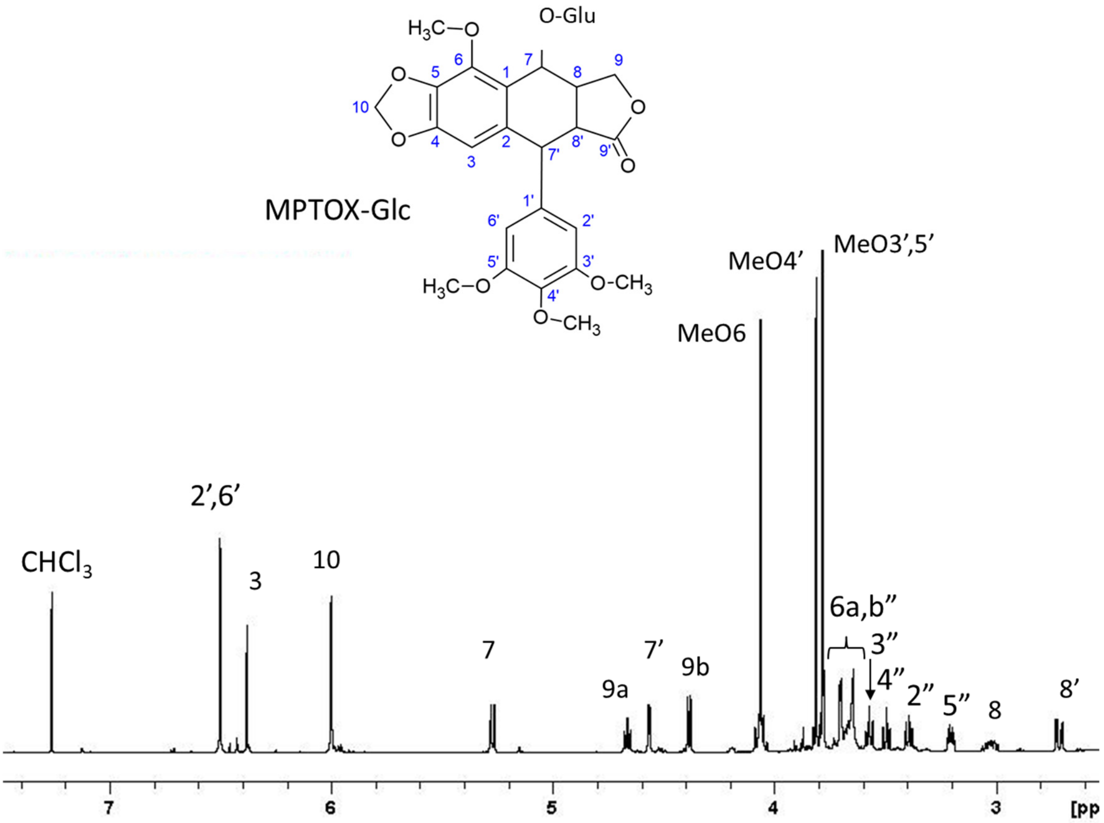

Figure S4 ^{1}H NMR spectra obtained from *L. flavum* sample with signals corresponding to 6-methoxypodophyllotoxin—7-O-β-glucoside

4. Statistical Report

Statistical analyses were performed using R, library rstatix, tidyverse, agricolae, corrr.

Two-way ANOVA of growth, phenols, flavonoids and antioxidant capacity

Residual analysis was performed to test for the assumptions of the two-way ANOVA. Outliers were assessed by box plot method; normality was assessed using Shapiro-Wilk's normality test and homogeneity of variances was assessed by Levene's test. There were no extreme outliers, residuals were normally distributed ($p > 0.05$) and there was homogeneity of variances ($p > 0.05$).

A two-way ANOVA was conducted to examine the effects of species and elicitor treatments on growth, accumulation of phenols, flavonoids and antioxidant capacity, as showed in **Table S1**.

Table S1 Two-way ANOVA of growth, phenols, flavonoids and antioxidant capacity

**Growth**					
Effect	DFn	DFd	F	p	η^2_g
Species	2	24	115.212	4.96e-13	0.906
Treatment	3	24	81.498	1.00e-12	0.911
Species x Treatment	6	24	18.200	7.41e-08	0.820
**Phenols**					
Effect	DFn	DFd	F	p	η^2_g
Species	2	24	112.748	6.28e-13	0.904
Treatment	3	24	402.706	1.19e-20	0.981
Species x Treatment	6	24	25.553	2.63e-09	0.865
**Flavonoids**					
Effect	DFn	DFd	F	p	η^2_g
Species	2	24	205.901	7.78e-16	0.945
Treatment	3	24	29.541	3.17e-08	0.787
Species x Treatment	6	24	5.128	2.00e-03	0.562
**Antioxidant capacity**					
Effect	DFn	DFd	F	p	η^2_g
Species	2	24	248.048	9.32e-17	0.954
Treatment	3	24	56.403	5.12e-11	0.876
Species x Treatment	6	24	8.863	3.79e-05	0.689

DFn, degrees of freedom in the numerator; DFd, degrees of freedom in the denominator; F, F-value; p, p-value; η^2_g, generalized eta squared.

4.1 Pairwise comparison of growth, phenols, flavonoids and antioxidant capacity

Tukey HSD post hoc tests were carried out. All pairwise comparisons were analyzed between the different species groups organized by elicitor treatments; the results are showed in **Figure S5** for growth, **Figure S6** for phenols, **Figure S7** for flavonoids and **Figure S8** for antioxidant capacity. Duncan test was performed considering $p < 0.05$.

Figure S5 Tukey HSD post hoc test for growth

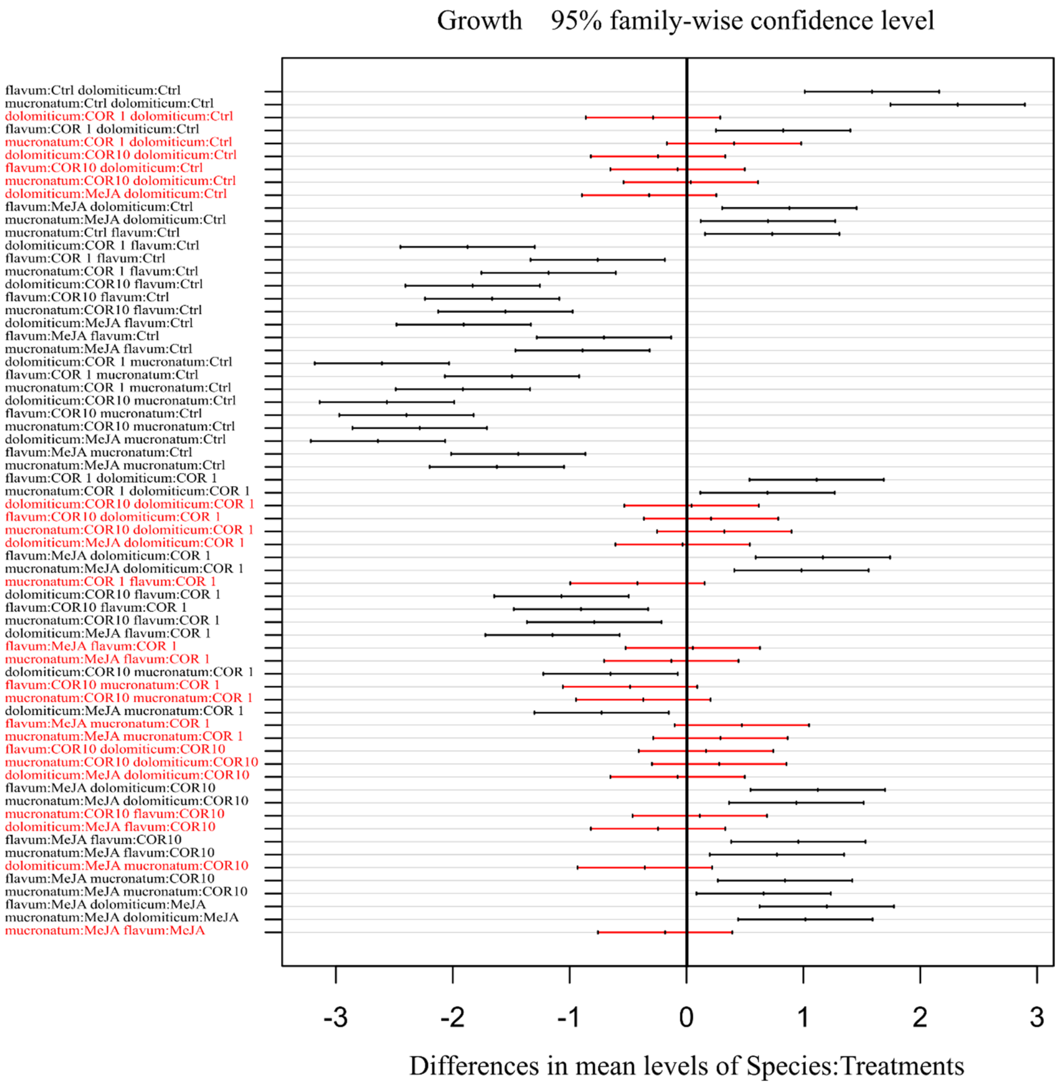

Figure S6 Tukey HSD post hoc test for phenols

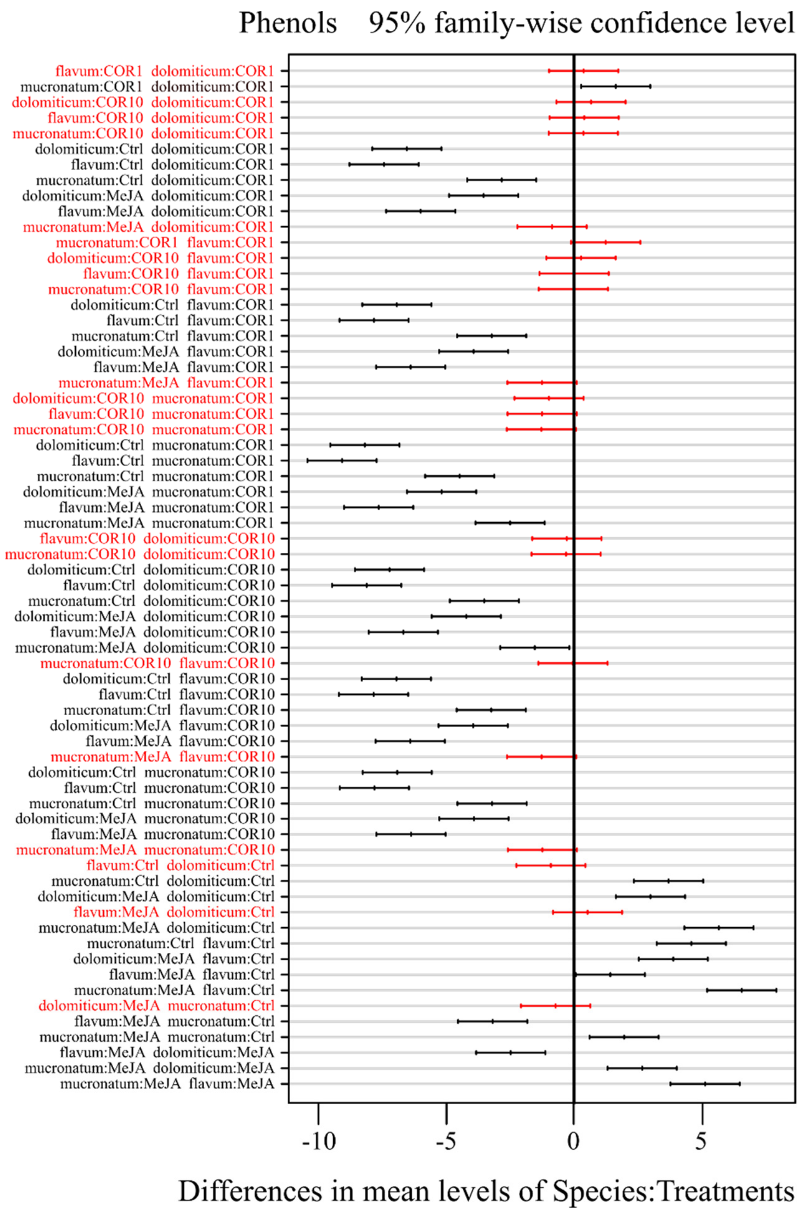

Figure S7 Tukey HSD post hoc test for flavonoids

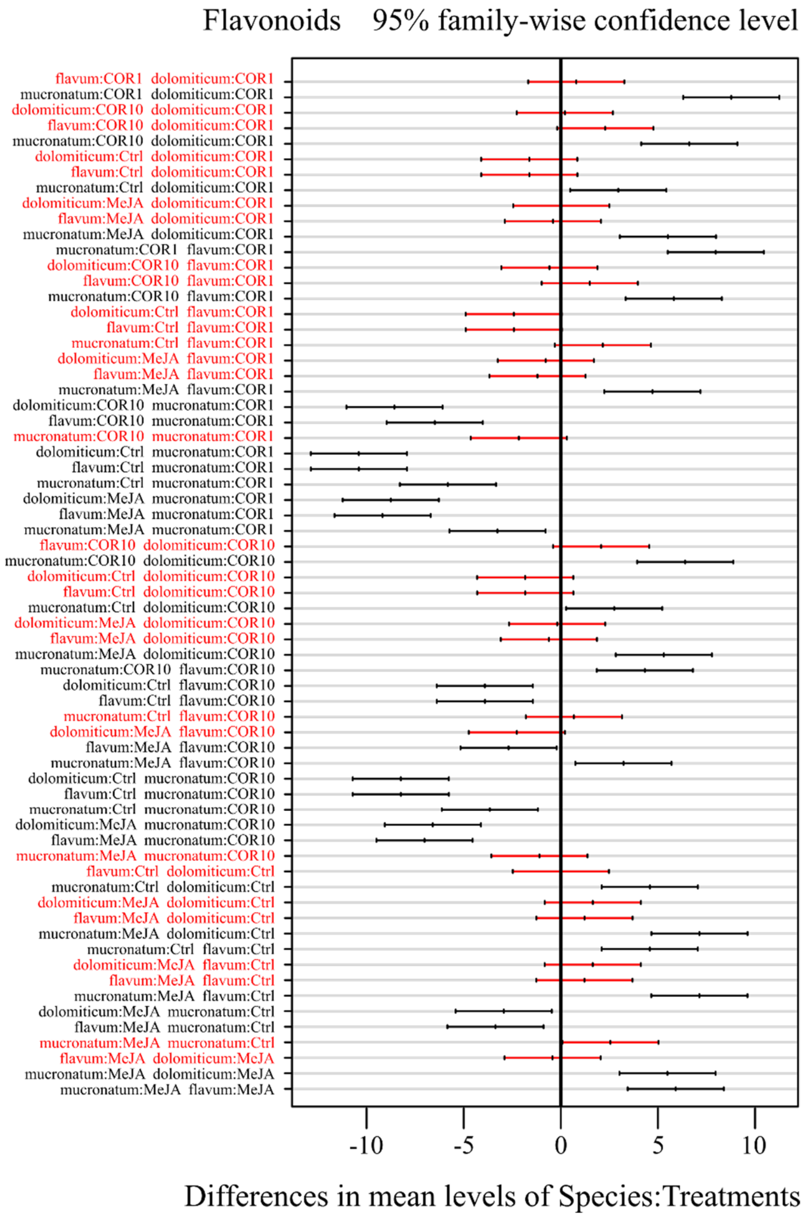

Figure S8 Tukey HSD post hoc test for antioxidant capacity

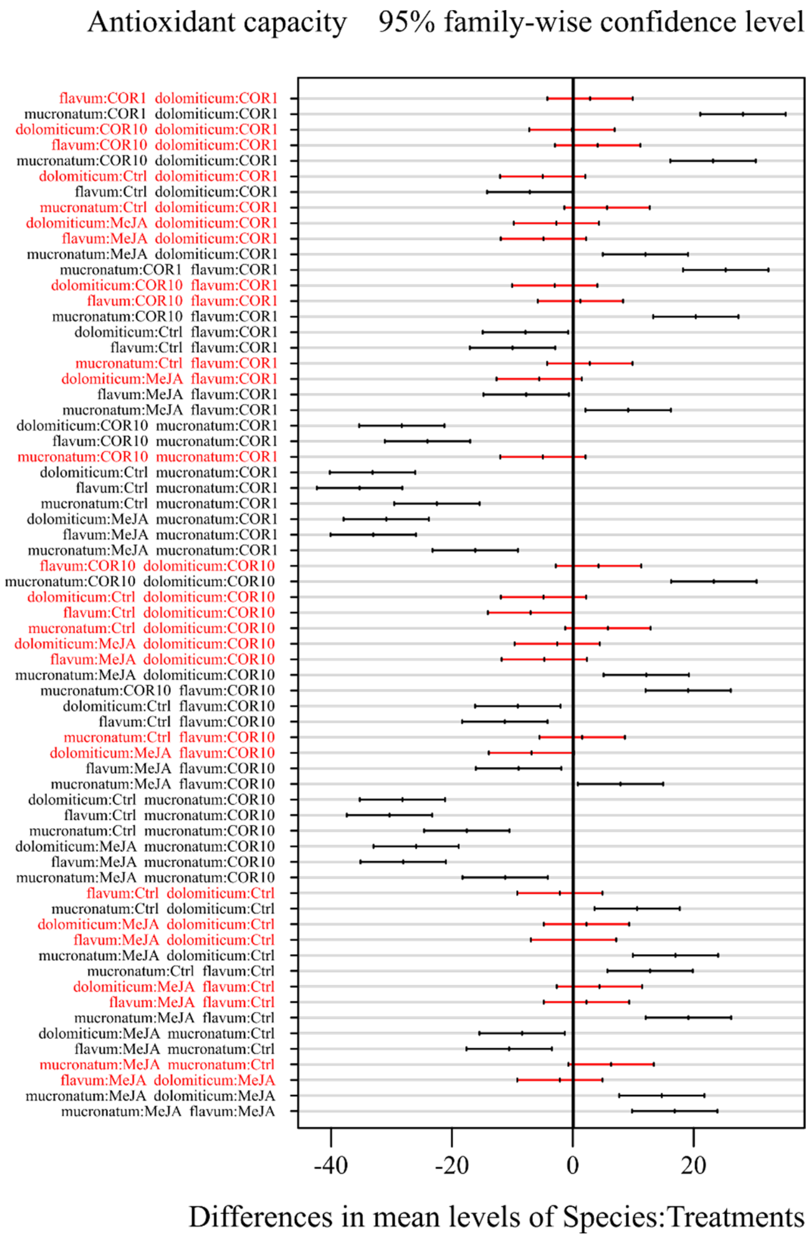

4.2 Two-way ANOVA on ATLs

Residual analysis was performed to test for the assumptions of the two-way ANOVA. Outliers were assessed by box plot method; normality was assessed using Shapiro-Wilk's normality test and homogeneity of variances was assessed by Levene's test. There were no extreme outliers,

residuals were normally distributed ($p > 0.05$) and there was homogeneity of variances ($p > 0.05$).

A two-way ANOVA was conducted to examine the effects of species and elicitor treatments on accumulation of PTOX, MPTOX and MPTOX—Glc, as showed in **Table S2.**

Table S2 Two-way ANOVA on ATLs

**PTOX**					
Effect	DFn	DFd	F	p	η_g^2
Species	1	16	55.553	1.37e-06	0.776
Treatment	3	16	138.832	1.15e-11	0.963
Species x Treatment	3	16	185.703	1.22e-12	0.972
**MPTOX**					
Effect	DFn	DFd	F	p	η_g^2
Species	2	24	61.943	3.34e-10	0.838
Treatment	3	24	25.125	1.40e-07	0.758
Species x Treatment	6	24	13.995	8.37e-07	0.778
**MPTOX-Glc**					
Effect	DFn	DFd	F	p	η_g^2
Species	2	24	335.469	2.88e-18	0.965
Treatment	3	24	2.682	6.90e-02, ns	0.251
Species x Treatment	6	24	7.655	1.14e-04	0.657

DFn, degrees of freedom in the numerator; DFd, degrees of freedom in the denominator; F, F-value; p, p-value; η_g^2, generalized eta squared

4.3 Pairwise comparison on ATLs

Tukey HSD post hoc tests were carried out. All pairwise comparisons were analyzed between the different species groups organized by elicitor treatments; the results are summarized in **Figure S9** for PTOX, **Figure S10** for MPTOX and **Figure S11** for MPTOX—Glc. Duncan test was performed considering $p < 0.05$.

Figure S9 Tukey HSD post hoc test for PTOX

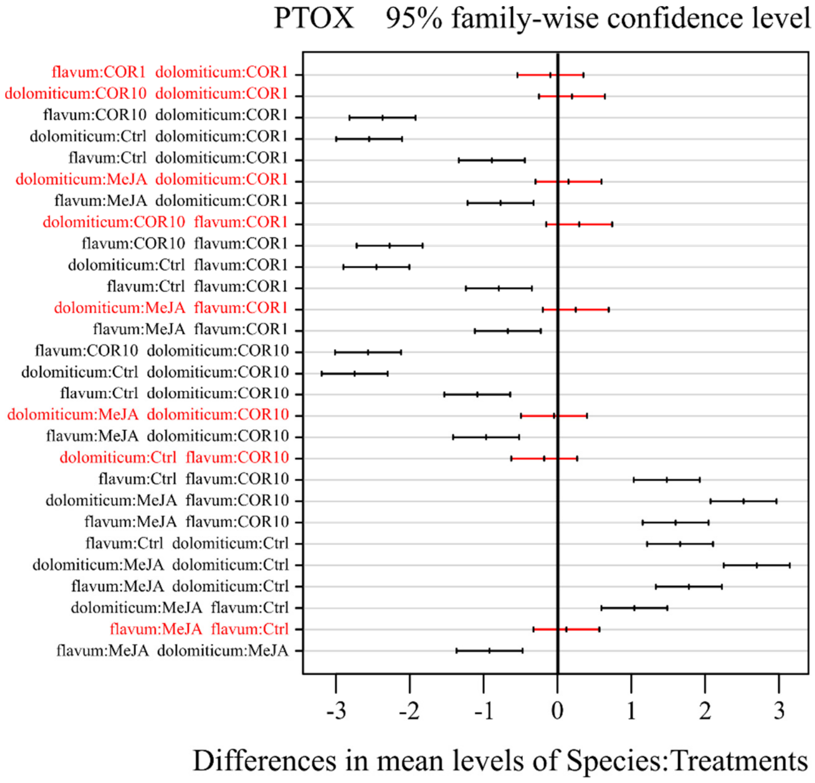

Figure S10 Tukey HSD post hoc test for MPTOX

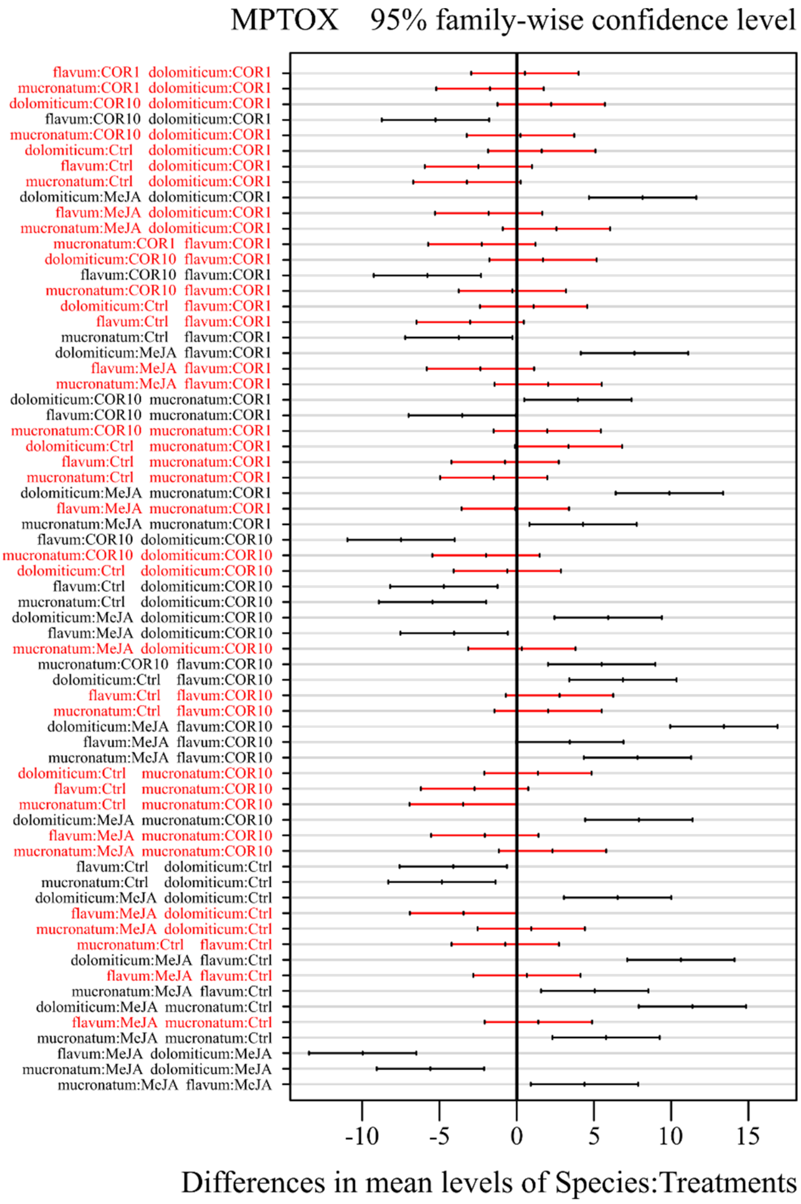

Figure S11 Tukey HSD post hoc test for MPTOX-Glc

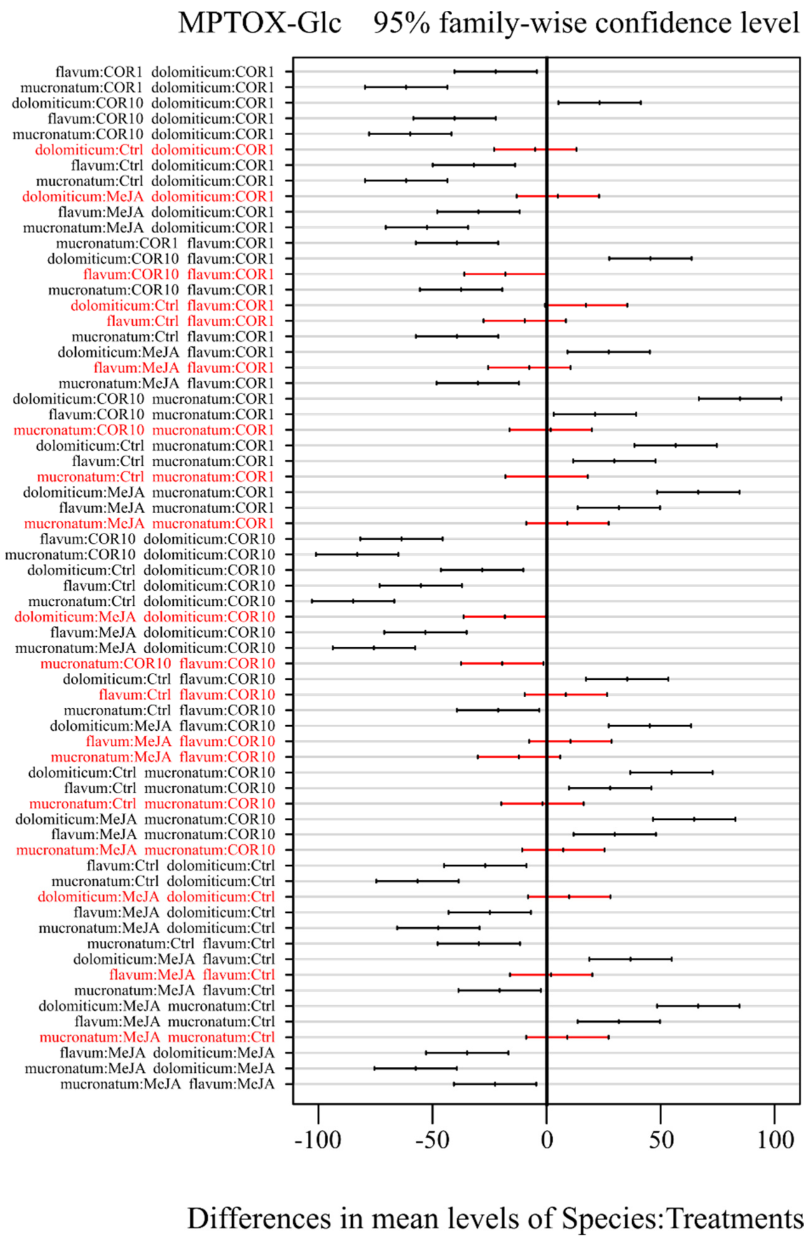

4.4 Pearson correlation

Figure S12 Correlation graphs for control (n = 9), MeJA (n = 9), COR 1μM (n = 9) and COR 10μM treated samples (n = 9). *, p ≤ 0.05; **, p ≤ 0.01; ***, p ≤ 0.001

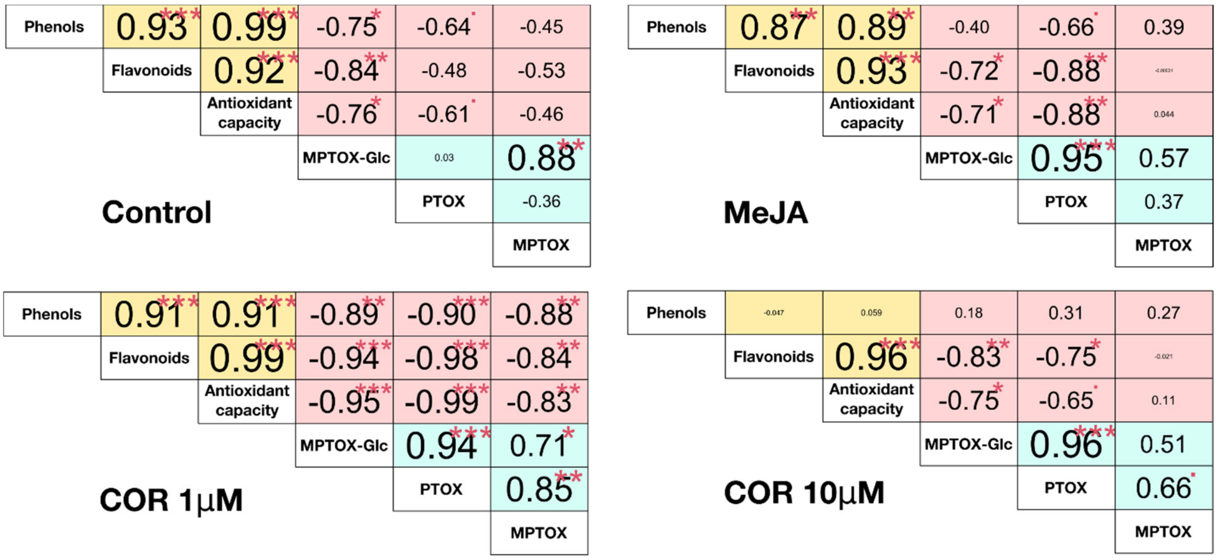

Supplement: Supplementary file 1 [file molecules-26-05189-s001.zip › molecules-1339780-supplementary.pdf]
